# Comparison of the Effect on Fetal Growth of a Mixture of Atrazine and Nitrates in Drinking Water and of Active Tobacco Exposure during Pregnancy

**DOI:** 10.3390/ijerph18042200

**Published:** 2021-02-23

**Authors:** Camille Carles, Marion Albouy-Llaty, Antoine Dupuis, Sylvie Rabouan, Virginie Migeot

**Affiliations:** 1 Univ. Bordeaux, INSERM UMR 1219, Equipe EPICENE, F33000 Bordeaux, France; 2 CHU de Bordeaux, Service Santé Travail Environnement, F33000 Bordeaux, France; 3 CHU Poitiers, Department of Public Health, F-86021 Poitiers, France; Marion.albouy@univ-poitiers.fr (M.A.-L.); virginie.migeot@chu-poitiers.fr (V.M.); 4 INSERM CIC 1402, CHU Poitiers, F-86021 Poitiers, France; antoine.dupuis@univ-poitiers.fr (A.D.); sylvie.rabouan@univ-poitiers.fr (S.R.); 5 School of Medicine and Pharmacy of Poitiers, Université de Poitiers, F-86073 Poitiers, France; 6 CHU Poitiers, Department of Pharmacy, F-86021 Poitiers, France

**Keywords:** endocrine-disrupting compounds, mixture, fetal growth, active tobacco exposure, drinking water

## Abstract

Active tobacco exposure during pregnancy is a known determinant of fetal growth. Nitrates and atrazine metabolites in drinking water may affect fetal growth as a mixture of endocrine disruptors (ED). We aimed to determine whether EDC have an additional effect on fetal growth compared to active tobacco exposure. A historic cohort study was carried out with a sample stratified with regard to the maternity unit, drinking water exposure, and year of birth. The women included were living in Deux-Sèvres, had given birth between 2005 and 2010 in three selected maternity units, and ultrasound data were available in their obstetrical records. Mixed linear models were used to analyze fetal weight evolution from the second trimester to the time of birth according to drinking water exposure to EDC mixture and active tobacco exposure. We included 558 mother-neonate couples, of whom 9% were exposed to high doses of the mixture and 21% to active tobacco smoking. There was no difference in fetal weight evolution according to drinking water mixture exposure (0.97 g; 95% CI [−3.01; 4.94]). We could not show a supplementary effect of mixture exposure in drinking-water on fetal growth as compared to active tobacco exposure. Further research is needed, using more precise methods to estimate EDC exposure.

## 1. Introduction

Adverse pregnancy outcomes such as low birth weight (LBW) and small-for-gestational-age (SGA) are known to have effects on neonatal and future adult health. More specifically, these outcomes may be associated with cardiovascular disease, type 2 diabetes, and psychomotor and cognitive impairment [1]. In addition, they are associated with higher rates of perinatal mortality [2].

Several factors are known to affect fetal growth [3]: genetic factors such as the infant’s gender and ethnic group; constitutional factors such as parents’ height and weight; socio-demographic factors including maternal age and parental income; obstetric factors involving parity, infertility, low birth weight in siblings, nutritional intake during pregnancy.

It is a known fact that active tobacco exposure is an established risk factor of perinatal outcomes, causing 24–25% of intrauterine growth retardation (IUGR) or SGA and 16% of LBW in developed countries [4,5]. Moreover, the effect of tobacco exposure can be observed as soon as the second trimester of pregnancy on head circumference and femur length [6]. Several hypotheses have been put forward on the effect of active tobacco exposure on fetal growth: 1: direct toxicity on fetal growth; 2: nutritional deprivation due to slowed blood circulation; 3: context of other risk factors for adverse pregnancy outcomes associated with maternal smoking during pregnancy [7,8].

While the second hypothesis is predominantly favored in the literature, the third hypothesis is getting growing attention. Tobacco smoke contains an endocrine disruptor mixture that could have antagonist or synergistic effects. Tobacco has an anti-estrogenic effect on women by inhibiting aromatase activity and by estradiol metabolite hydroxylation [9]. Lower levels of estradiol are measured in newborns exposed to active tobacco during pregnancy. In addition, three pesticides of the dinitroaniline family have been detected in tobacco smoke [10]. These three pesticides are known or suspected endocrine disruptors. However, tobacco smoke contains a lot of other pollutants such as carbon monoxide which could also affect fetal growth by impairing oxygen transport [7].

Drinking water exposure to environmental pollutants has also been studied, and associations between drinking water exposure to endocrine disruptors and SGA or LBW have been discovered: mothers exposed during pregnancy to atrazine [11,12,13,14,15] or a mixture of atrazine metabolites and nitrates [16] in drinking water were more likely to give birth to SGA or LBW babies.

Nitrates are potential Endocrine disruptive compounds (EDCs) with an anti-androgenic effect [17,18] by inhibiting the steroid hormone syntheses via conversion to nitric oxide. Despite its effect on oxygen transport by induction of methemoglobin, nitric oxide inhibits the cytochrome P450 enzymes stopping the transformation of free cholesterol into progesterone [19]. Atrazine is likewise a potential EDC with anti-androgenic and weak estrogenic effects [20]. There may therefore exist an additive effect of active tobacco exposure when mixed with environmental exposure to EDC during pregnancy. Indeed, EDCs have specific properties such as synergism or antagonism when mixed, which depend on the dose [21] and on each substance’s mode of action [22]. In the literature, most studies have highlighted the effect on fetal growth of a single compound (tobacco, pesticides), while two studies have focused on a mixture exposure of atrazine metabolite and nitrate [16,23].

A dose-response relationship between active tobacco exposure and fetal growth has been repeatedly observed [4,24,25], suggesting a causal relationship between tobacco exposure and fetal growth. The effect of maternal tobacco smoking on fetal growth has been described as early as 18 weeks of gestation (WG) on ultrasound biometry measurements and could vary according to the time period of pregnancy, thereby introducing the notion of window exposure effect on fetal growth [25,26]. Definitions of outcomes in the literature have nonetheless differed (low birth weight, below 2500 g, SGA and IUGR) as have the exposure periods studied (entire pregnancy, second trimester, third trimester). All of them have used fetal growth measurements at birth, particularly birth weight, the usual indicator of fetal growth. Because of these discrepancies, no period of greater vulnerability of the fetus or exposure window during pregnancy has been found. However, fetal growth can be assessed early in pregnancy with ultrasound measurements: biparietal diameter (BPD), abdominal circumference (AC), and femur length (FL). These biometry parameters can be used to calculate estimated fetal weight (EFW), according to mathematical formulas [27]. Biometry parameters enable searching for exposure effects during pregnancy, before birth, and at specific times, according to the exposures of interest.

We aimed to determine whether exposure to endocrine-disrupting compounds in drinking water has an additional effect on fetal growth measured by fetal weight compared to the known and observed effect of active tobacco exposure during pregnancy.

## 2. Materials and Methods

### 2.1. Study Area and Timeline

This retrospective cohort study was conducted between 2005 and 2010 in Deux-Sèvres, in western France. In this district of 5999 km² with 362,944 inhabitants in 2007, agricultural activity is predominant with 75% of land use essentially involving livestock, mainly sheep and goats along with cereal production. We chose Deux-Sèvres because of its rurality and consequently sizable use of pesticides and also because of the highest known ground concentration of nitrates in the western part of France.

### 2.2. Data Collection

The French regional health agency (ARS, Agence Régionale de Santé) regularly assesses pesticide and nitrate levels in drinking water. As required by law, the number of measurements by the municipality is proportional to population size. Municipalities are split or grouped into community water systems (CWS), geographic areas receiving drinking water from the same source, and water quality is considered to be homogeneous within a CWS. All the samples concerned treated water, and were taken from CWS and water treatment plants, between 1 April 2004 and 31 December 2010 to obtain complete exposure data throughout the pregnancy of every woman included in the study.

Birth records are drawn from the infant health certificates issued by the district office of maternal and childhood protection. Completed by the hospital or clinic before the infant’s discharge, these documents are mandatory, and thereby likely to include practically all births. The information contained in these certificates includes sex, birth weight, gestational age (weeks of gestation at birth), age of mother, number of previous pregnancies, parental occupation, and place of residence at birth. Data from birth records were validated according to a methodology approved by the French “Direction de la recherche, des études, de l’évaluation et des statistiques” [28].

### 2.3. Population Study

We identified all live births in Deux-Sèvres from 1 January 2005 to 31 December 2010 of neonates whose mothers lived in the district at the time of birth, whose birth took place in the maternity units of the hospitals of Niort, Poitiers, and Bressuire, and whose birth certificate had been recorded. We excluded the non-environmental causes known to induce low birth weight such as multiple births, early deaths (before birth record completion), neonates with congenital abnormalities, and birth by cesarean section. We also excluded neonates whose mothers lived in municipalities having more than one CWS providing drinking water or whose place of residence could not be identified. Sampling date, sampling location, and CWS or treatment plant names were available for each measurement in drinking water. Concentrations of nitrates and atrazine in drinking-water routinely measured in drinking-water were assigned by the maternal municipality of residence at birth. These concentrations, considered as a proxy of EDC exposure in drinking-water were merged with birth and obstetrical records by the place of residence of the mother at birth.

Because information about pregnancy ultrasounds and certain maternal characteristics are not available in birth records, we went to maternity units to collect supplementary data in the obstetrical records. Because of organizational and time constraints, we selected a random sample of mother-neonate couples, stratified with regard to year of birth, exposure status to atrazine metabolites during pregnancy, and maternity unit of birth.

A number of subjects were measured to show a difference of 100 g of fetal weight at the third trimester between couples exposed and couples not exposed to atrazine metabolites for an α-risk of 5% with a power of 80%. The choice of the difference was based on two of the quantified effects of environmental exposure on EFW at third trimester found in the literature: active tobacco exposure with a difference in EFW of 56 g [8] and outdoor air pollution exposure with a difference in EFW of 200 g [29]. In the absence of other data in the literature, we chose between the values of the effect on fetal growth of active tobacco exposure (56 g) and air pollution exposure (200 g) and chose a value of 100 g. The number of subjects needed was 674 couples and we decided to add 20% of the number of subjects needed in our sample to take account of potential missing data, which lead to a final number of 800 couples.

Data about ultrasounds, gestational diabetes, smoking during pregnancy and maternal weight and height were obtained in the obstetrical records of the mothers sampled. At this step, we excluded mother-neonate couples whose files did not contain detailed ultrasound reports from the second and third trimester and cases in which ultrasound measurements were carried out before 20 or after 25 weeks of gestation (WG) for the second trimester and before 30 and after 35 WG for the third trimester. We also excluded births occurring before 35 WG so that there was no overlap of gestational age between estimated fetal weight at third trimester and birth weight.

**Figure 1:**
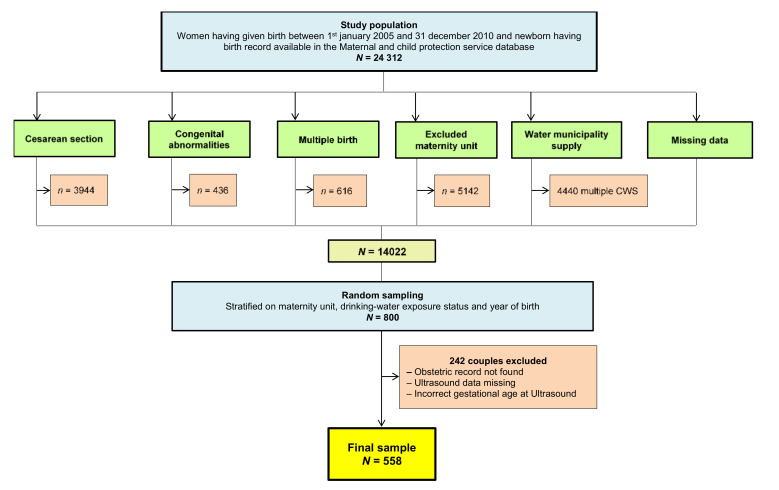
Flow chart, Deux-Sèvres, France. (CWS: Community water system).

Among the study population of 14,022 mother-neonate couples, 800 were randomly sampled and 558 were finally included in the study, as shown in the flowchart (Figure 1).

### 2.4. Health Outcomes

Our principal outcome consisted of the evolution of fetal weight between second trimester and birth (repeated measurements for each subject at second, third trimester, and birth). Estimated fetal weight at second and third trimesters of pregnancy was calculated by a mathematical formula based on fetal biometry parameters: biparietal diameter (BPD), abdominal circumference (AC), and femur length (FL), given by the results of pregnancy ultrasounds. This formula was developed by Hadlock [30]: Log10(PFE) = 1.335 + 0.0316.BPD + 0.0457.AC + 0.1623.FL − 0.0034.AC.FL. Birth weight was collected in birth records.

### 2.5. Exposure Assessment

Two types of exposure during pregnancy were studied: drinking water exposure to a mixture of atrazine metabolites (pesticides most often found in drinking water) and nitrates defined according to a method described elsewhere [16] and active tobacco smoking of the mother during pregnancy. Chemical analyses in drinking-water are mandatory in France and are done by accredited laboratories that are certified by the ministry of health according to the law [31], providing the technical and administrative conditions of the analyses. These laboratories are then selected by regional health agencies (ARS) by invitation to tender. Sampling on site is carried out by ARS agents or by certified lab technicians, and sampling locations are selected according to hazards identified in the drinking-water production or distribution system.

Mixture exposure was defined by six classes (unexposed to pesticides and exposed to the first tercile of the mean concentration of nitrates; unexposed to pesticides and exposed to the second tercile of the mean concentration of nitrates; unexposed to pesticides and exposed to the third tercile of the mean concentration of nitrates; exposed to pesticides and first tercile of the mean concentration of nitrates; exposed to pesticides and the second tercile of the mean concentration of nitrates; exposed to pesticides and the third tercile of the mean concentration of nitrates). Active tobacco exposure during pregnancy was defined as a binary variable: yes/no.

### 2.6. Statistical Analyses

The following analyses were weighted on the inverse of sample probabilities according to the stratification used to correct an overrepresentation of mother-neonate couples exposed to atrazine and its metabolites in drinking-water in our sample and calculated fetal weight evolution during pregnancy in grams and 95% confidence intervals (95%CI). Covariates were selected in the multivariate analysis according to three criteria: they were significantly associated with the outcome and the exposure variables at a threshold of 25%; they were known or suspected confounding factors in the literature; they were independent of each other. They included covariates acting on birth weight: newborn gender; maternal weight before pregnancy (in kilograms); maternal age (<27; 27–29; 29–33; >33 years); the history of low birth weight in siblings (yes/no); household occupation (disadvantaged: workers and unemployed; moderately advantaged: self-employed, employees and farmers; advantaged: managers and executives); gestational diabetes; gestational age at ultrasound examination or birth (weeks of gestation) and factors influencing drinking water exposure: rural location of residence at birth; season during which the second trimester of pregnancy took place. We included rural and seasonal variables in the analyses because pesticide usage is predominant in summer and autumn and rural areas.

Our outcome, fetal weight evolution between second trimester and birth was studied by longitudinal data analysis using linear mixed models. The linear mixed models take account of intra-subject correlation caused by repeated data by using the variance of random-effects parameters as covariance parameters. The slope of the models was the evolution of gestational age between each fetal weight measurement. We used three linear mixed models: one model with both exposures (model #1), another model with only drinking water exposure to the mixture of atrazine metabolites and nitrates (model #2), and a third model with only active tobacco exposure (model #3). The outcome was modeled as the exponential of the fetal weight evolution in grams. Random effects on intercept and slope (weeks of gestational age) were allowed. The goodness of fit was assessed by consideration of independence and normality of the residuals [32]. To fulfill the aim of our study, we tested the interaction terms between EDC mixture exposure and active tobacco exposure during pregnancy and we compared nested models using the likelihood ratio (LR) test and non-nested models using the Akaike criteria (AIC).

Analyses were performed with SAS using the proc mixed procedure (version 9.3; SAS Institute, Cary, NC, USA).

## 3. Results

Among the 558 mother-neonate couples, the average number of atrazine metabolite measurements in drinking water during pregnancy was 3 ± 1 and the average number of nitrate measurements was 35 ± 30.

During the study period, 438 drinking water atrazine metabolite measurements were carried out; for desethylatrazine and 2-hydroxyatrazine (the most frequent metabolites) they ranged from 0 to 0.1 µg/L. Most of them (432, 98.4%) came from treatment plant water. During pregnancy, 283 mothers (corresponding to 50.7% of the raw total and 42.5% of the weighted total) were exposed to atrazine metabolites with an average concentration of atrazine metabolites in drinking water of 0.04 ± 0.02 µg/L for both desethylatrazine and 2-hydroxyatrazine. During the study period, 3168 drinking water nitrate measurements were performed; they ranged from 0 to 63.3 mg/L and came from CWS waters only. During pregnancy, all mothers were exposed to nitrates with an average concentration of nitrates in drinking water of 23.5 ± 11.8 mg/L. Limits of drinking water nitrate concentration terciles were 18.1 and 30.3 mg/L during the entire pregnancy.

Study population characteristics are presented in Table 1. The mean estimated fetal weight was 558.2 ± 4.8 g at the second trimester and 2068.6 ± 14.0 g at the third trimester. Mean gestational age at completion of ultrasound measurement was 22.3 ± 0.04 weeks of gestation (WG) at the second trimester and 32.3 ± 0.04 WG at the third trimester. Mean birth weight was 3337.3 ± 23.5 g and mean gestational age at birth was 39.4 ± 0.1 WG. Before adjustment on the available confounders, fetal weight evolution between second trimester and birth was associated with active tobacco exposure during pregnancy but not with drinking water exposure to an atrazine metabolite and nitrate mixture (Table 2). After adjustment on the available confounders, fetal weight evolution between second trimester and birth was not associated with drinking water exposure to an atrazine metabolite and nitrate mixture. However, fetal weight evolution was associated with tobacco exposure with (−3.46 g per WG [−6.07; −0.85]) or without drinking water exposure to an atrazine metabolites and nitrates mixture (−3.43 g per WG [−6.05; −0.82]) (Table 2 and Table 3). The interaction terms between EDC mixture exposure and tobacco smoking during pregnancy were not significant (F test = 0.58, *p* = 0.717). Comparing both models, the likelihood ratio test was not significant (LR khi² test = 6.6, *p* = 0.25).

## 4. Discussion

Our results did not show that drinking water exposure to an EDC mixture has an additional adverse effect on fetal growth between the second trimester and birth when active tobacco exposure is likewise taken into consideration. We found a decrease in fetal weight evolution between the second trimester of pregnancy and birth when the mother smoked during pregnancy (−3.46 g per week of gestation (WG) [−6.07; −0.85]). The decrease in fetal weight evolution during pregnancy of 3.46 g per WG represents a difference of 138 g at birth (40 WG) between exposed and unexposed neonates. This result is consistent with the literature. Jaddoe and al [25] observed a difference of 200 g at birth between neonates actively exposed and neonates not exposed to tobacco during pregnancy, and Gaillard and al noted a difference of 165 g at birth (40 SA) [33]. More recently, Cardenas et al. [34] observed a difference of 175 g at birth between exposed and unexposed neonates. Furthermore, they observed that prenatal maternal smoking might interact with placental DNA methylation at specific loci in the epigenome, mediating the association with lower birth weight in infants.

We did not find a significant association between fetal weight evolution and drinking water exposure to atrazine metabolites and nitrate mixture. Almberg et al. [11] observed an increased risk of low birth weight associated with atrazine exposure in drinking water over the entire gestational period OR 1.27 1.10–1.45) and the first (OR 1.20 1.08–1.34) and second trimester (OR 1.13 1.07–1.20) of pregnancy. However, no association was observed between SGA and atrazine exposure in drinking-water. But atrazine concentrations in the Almberg study were higher, ranging from 0 to 15 µg/L with geometric annual means of 0.15, 0.16, and 0.29 µg/L, which could explain why results were inconsistent.

The study population came from birth records that may be considered exhaustive as they are mandatory in France. Sample selection was stratified on drinking water pesticide exposure status according to the results of a prior study [16], year, and maternity ward of birth. Stratification on year and maternity ward of birth was carried out with equal probability. We stratified on the year of birth to take account of pesticide level variations between locations and over the years as atrazine and its metabolites are more present in drinking water in the district of Deux-Sèvres [35] and since in Europe atrazine has been forbidden from sale since 2002 and from use since 2003 [36]. Therefore, residual atrazine is still present in drinking-water but its concentration may decrease over time. We stratified on maternity unit of birth for feasibility reasons. Mother-neonate couples exposed to pesticides were over-represented in the sample because exposure prevalence in the population is low. Stratification was taken into account in the analysis by weighting on sample probability and stratifying on birth year according to a method described elsewhere [37]. Any selection bias was thereby avoided. Our study population is representative of the general French population in 2010, particularly with regard to the prevalence of mothers smoking during pregnancy [38].

We studied the effects of a mixture exposure of atrazine metabolites and nitrates, both of them endocrine-disrupting compounds that have specific properties such as dose-response [39] and synergistic effects [21] when mixed. We did not study the effect of drinking water exposure to atrazine metabolites or nitrates alone because exposure to only one EDC does not reflect reality and consequently does not seem relevant [16]. Assessment of drinking-water exposure is very limited in birth cohorts. Indeed, water contaminants have received the least attention among all other environmental risks in the literature. However, pregnant and lactating women may consume more drinking water than non-pregnant women, increasing their daily intake of water contaminants [40].

A major strength of our study consists in its repeated measurements of fetal biometry, which facilitate observation of effects at different stages of pregnancy and consequently allow for detailed study of fetal development over time using longitudinal models and taking inter-individual variability and intra-individual correlation into close consideration. To our knowledge, there exist only a few studies using a similar approach [8,41,42,43,44] but none of them have analyzed the effects of presence or absence of active tobacco exposure along with drinking water exposure to an EDC mixture. Unfortunately, such a method implies that the exposure effect on fetal growth is homogeneous during pregnancy and the result is averaged, without considering potential time exposure windows [45] when the fetus could be more vulnerable and likewise without considering fetal growth physiology [46] with a greater weight gain at third trimester. It may induce misclassification of exposure and underestimation of the association when an actual window of exposure exists.

Our work has some limitations. The scarcity of significant results in our study may be due to a lack of power. We chose a sample size based on the results of the effect of active tobacco exposure [8,47] and outdoor air pollution exposure [29] on fetal growth during pregnancy even though, to our knowledge, there exists no study quantifying the effect on fetal growth of drinking water exposure to pesticides and nitrates, taken either separately or in a mixture. And since at present there is no widely accepted general standard for sample size computations in mixed linear models presenting both fixed and random effects, it was with the method used for multiple linear models that we determined the sample size. Moreover, our number of subjects, in the final analysis, was much lower than expected because of missing data and could have also lead to a significant lack of power which could have a substantial effect on our results.

Our assessment of drinking water exposure may not reflect the actual exposure of pregnant women. Laboratory analyses of water quality are done to verify whether or not concentrations are above the regulatory limit, which is largely above the detection limit. Therefore, they do not search for the detection limit, only for the quantification limit. In our study, the data situated between detection and quantification limits were considered as the absence of exposure to atrazine metabolites. It is possible that we underestimated exposure to atrazine metabolites and subsequently to a mixture and that conversely, we overestimated the association between mixture exposure and fetal growth. Furthermore, drinking water exposure depends on drinking water consumption patterns. In the western part of France, where the district of Deux-Sèvres is located, the percentage of people drinking tap water was estimated in 2007 at 61% [48]. In the United States, it has been observed that pregnant and non-pregnant women do not differ in tap water consumption [49]. Furthermore, recent results from the Endocrine Disruptor Deux-Sèvres (EDDS) cohort study showed that 71% of pregnant women drank tap water [50] so it may not have affected our estimates. Besides, using ecological data on drinking-water prevents selection bias that can be found in cohort studies. Moreover, ecological studies allow one to study large populations and therefore provide greater statistical power. They also use existing databases, which can be used directly without the necessity of contacting a large population [51,52]. This design appears to be a very cost-efficient epidemiological approach and can be executed in a relatively short period of time and can cover large territories in terms of environmental exposure.

Other measurement methods could have been used to assess EDC mixture exposure such as declarative data collected by questionnaire, individual drinking-water metrology, or biometrology in urines, blood, or breast milk. All these methods bring individual assessment, but declarative data is based on personal recall and may lead to a misclassification of exposure. Individual metrology of drinking water, in the home of the mothers, could be useful but requires to go to the homes of each couple, which is costly and complicated in terms of organization and logistics. At last, biometrology is a very effective way to assess EDC mixture exposure but requires the consent of each mother and also leads to practical difficulties and costs in our study.

In the birth records placed at our disposal, no data were available on women’s mobility during pregnancy. All we had at our disposal was the mother’s home address at the time of birth. However, moving during pregnancy occurs in only 9–32% of cases, mostly during the second trimester and for a short distance (<10 km) [53,54] so we can assume that it did not significantly affect our exposure estimates.

Our exposure assessment was not an individual estimation of exposure, which nonetheless seems to be the best method, by avoiding selection bias. EDC exposure assessment can be rendered more precise through exposure biomarkers such as blood and urine or cord blood, amniotic fluid, and breast milk, with concentrations that could reflect fetal exposure [12,55]. For example, BPA and its chlorinated derivatives have been successfully detected and reliably quantified in human urine and colostrum, and the methods applied can be used to estimate fetal exposure to these EDC in drinking water [56,57]. Moreover, BPA and phthalate urinary levels have been combined with ultrasound measurements of fetal growth in the literature [58]. Atrazine is particularly lipophilic and accumulates in mammary tissues in rats, according to the amount of dose administered and transferred to the offspring via milk [59]. However, temporal and spatial variability and the limited samples of the individual estimation method [55] show that ecological assessment, although less accurate, is still useful. Moreover, urinary atrazine metabolites were significantly correlated to tap water consumption during pregnancy [60].

Data on mother-neonate couples were limited to the information available on birth and obstetrical records. Smoking status during pregnancy was binary (yes/no). We did not have enough information in obstetrical records on the number of cigarettes smoked so we could not evaluate the dose of active tobacco exposure. But most other studies have likewise compared smoking to non-smoking mothers and focused on the exposure window [8,26,47]. Another limitation of our study consists in the fact that data on active smoking during pregnancy was declarative, which may have induced misclassification of exposure and an under or overestimation of the association. The ultrasound measurements that we used to build our outcome allow for the study of fetal growth early in pregnancy, well before birth, and therefore for earlier detection of issues. Other studies assessing the association between drinking water pesticide or nitrate exposure and fetal growth have applied indicators of the latter at the time of birth [11,14,15,16,61], not earlier in pregnancy.

We did not have information on the ultrasound operators, the measurement methods, or the machines used because this data was not available in the obstetrical records. The measurement variability derived from this lack of information could decrease the precision of our estimations. But the literature shows a good inter and intra-observer reproducibility [62,63], so the lack of precision in our estimations of fetal weight should be limited. We could not use a formula with all biometry parameters because of the unavailability of obstetrical records of ultrasound measurements of head circumference. We chose Hadlock’s formula with three parameters of biometry to determine EFW [27]. Even though the equation was obtained from data based on a small number of subjects and although estimation becomes less precise with distance from the term, this formula has the same mean absolute error as the formula involving all parameters [64]. Estimation of fetal weight at any gestational age would be more precise with magnetic resonance imaging and volumetric equations [62] but access to this highly specific technique is distinctly limited in current practice. Nevertheless, the formula used in this study is among the most precise ones available and also one of the most widely used in clinical practice [64,65].

Atrazine exposure was measured only in drinking water, which meant that airborne or food exposure could not be assessed. However, it has been shown that atrazine metabolites are rarely found in food and that their atmospheric concentration is usually quite low, just above the detection limit [66]. We did not take into account environmental tobacco exposure, but this factor does not modify the association between active tobacco exposure and prevalence of small for gestational age or low birth weight newborns [67] nor is it associated with fetal growth earlier in pregnancy [8]. We lacked data on maternal alcohol consumption and nutritional behavior, factors that can also have an effect on fetal growth [3]. But the available data was of attested good quality, having been subjected to quality control with the validated procedure.

A previous study with an ecological estimation found an association between EDC mixture exposure during pregnancy and birth weight [16] but this study was based only on existing data and the sample of mother-neonate couples was much larger, limiting the lack of power, unlike our current work. Although it was not observed earlier in pregnancy, a possible effect of EDC mixture on fetal growth may exist, and it should help to motivate preventive actions towards pregnant women on EDC [68].

## 5. Conclusions

This historical cohort study did not show that drinking water exposure to an EDC mixture may have an additional adverse effect on fetal growth between the second trimester and birth in the event of active tobacco exposure, even though previous works had found that an EDC mixture may affect birth weight. Future studies are needed, with larger samples and using endocrine-disrupting compound biomarkers in blood and breast milk associated with a more individualized assessment of drinking-water exposure to provide more precise and unbiased fetal exposure estimates. Besides, as tobacco smoking and other toxic factors such as EDC mixture exposure may have associated effects on fetal growth, future studies should investigate exposure to more than one toxic factor on fetal growth. Furthermore, to better understand fetal growth dynamics, more complex methods such as prenatal growth curves could constitute valuable modeling, taking into account an appreciable number of fetal and parental characteristics.

## Figures and Tables

**Table 1:** Characteristics of the study population and potential confounders, Deux-Sèvres, France.

Characteristics of The Study Population and Potential Confounders	Raw Results				Weighted Results *	
	Missing Values		Total (*N* = 558)		Total (*N* = 9013)	
	*N*	%	*N*	%	*N*	%
Sex of the newborn	*0*	*0*				
Boy			238	42.7	3893	43.2
Girl			320	57.4	5120	56.8
Premature birth	*0*	*0*				
Yes			16	2.9	282	3.1
Household occupation	*5*	*1*				
Disadvantaged			69	12.5	1157	13.0
Moderately advantaged			352	63.7	5932	66.5
Advantaged			132	23.9	1825	20.5
Rural location of residence	*0*	*0*				
Yes			206	36.9	2974	33.0
Primiparity	*13*	*2*				
Yes			215	39.5	3329	37.6
Maternal age (years)	*0*	*0*				
<27			135	24.2	2211	24.5
27–29			124	22.2	2144	23.8
30–33			154	27.6	2497	27.7
>33			145	26.0	2161	24.0
Maternal body mass index (kg/m²)	*6*	*1*				
<18			24	4.4	395	4.5
18–24			376	68.1	5858	66.1
25–29			91	16.5	1605	18.1
>29			61	11.1	1011	11.4
History of low birthweight	*7*	*1*				
Yes			20	3.6	314	3.5
Gestational diabetes	*49*	*9*				
Yes			42	8.3	685	8.3
Season during second trimester	0	0				
Spring			124	22.2	2131	23.6
Summer			146	26.2	2214	24.6
Fall			132	23.7	2193	24.3
Winter			156	28.0	2475	27.5
Smoking during pregnancy	*50*	*9*				
Yes			110	21.7	1665	20.5
Mixture exposure in drinking water during the whole pregnancy	*0*	*0*				
Atrazine metabolites No and Nitrates < 18.14 mg/L			66	11.8	1224	13.6
Atrazine metabolites No and Nitrates 18.14–30.33 mg/L			137	24.6	2679	29.7
Atrazine metabolites No and Nitrates > 30.33 mg/L			72	12.9	1279	14.2
Atrazine metabolites Yes and Nitrates < 18.14 mg/L			120	21.5	2532	28.1
Atrazine metabolites Yes and Nitrates 18.14–30.33 mg/L			54	9.7	538	6.0
Atrazine metabolites Yes and Nitrates > 30.33 mg/L			109	19.5	761	8.4

* Weight defined by the inverse of the sample probabilities.

**Table 2:** Fetal weight evolution during pregnancy according to drinking water mixture exposure and active tobacco exposure before and after adjustment for available confounders, Deux-Sèvres, France.

Fetal Weight Evolution during Pregnancy According to Drinking Water Mixture Exposure and Active Tobacco Exposure before and after Adjustment for Available Confounders	Fetal Weight between Second Trimester and Birth ^&^ in Grams					
	Unadjusted Analysis			Adjusted Analysis (*N* = 458) Model #1		
	Difference	95%CI	*p*	Difference	95%CI	*p*
Weight evolution according to drinking-water mixture exposure during pregnancy in grams			0.512			0.481
Atrazine metabolites No and Nitrates < 18.14 mg/L	1			1		
Atrazine metabolites No and Nitrates 18.14–30.33 mg/L	−0.60	[−4.48; 3.28]		−0.76	[−4.56; 3.04]	
Atrazine metabolites No and Nitrates > 30.33 mg/L	−1.48	[−6.03; 3.06]		−1.60	[−6.06; 2.86]	
Atrazine metabolites Yes and Nitrates < 18.14 mg/L	1.98	[−2.03; 5.99]		1.85	[−2.08; 5.79]	
Atrazine metabolites Yes and Nitrates 18.14–30.33 mg/L	0.02	[−4.62; 4.66]		−0.17	[−4.72; 4.38]	
Atrazine metabolites Yes and Nitrates > 30.33 mg/L	1.06	[−2.99; 5.11]		0.97	[−3.01; 4.94]	
Weight evolution according to smoking during pregnancy in grams			0.010			0.009
No	1			1		
Yes	−3.48	[−6.13; −0.83]		−3.46	[−6.07; −0.85]	
Season during the second trimester			0.812			0.759
Summer	1			1		
Fall	−29.66	[−90.53; 31.21]		−31.37	[−91.21; 28.48]	
Winter	−15.13	[−73.63; 43.37]		−20.92	[−79.78; 37.94]	
Spring	−19.46	[−82.84; 43.91]		−10.22	[−72.78; 52.34]	
Newborn gender			0.004			0.005
Boy	1			1		
Girl	−64.62	[−108.46; −20.78]		−62.26	[−105.98; −18.55]	
Maternal weight before pregnancy (kg)	2.65	[1.09; 4.22]	0.001	2.48	[0.91; 4.05]	0.002
Maternal age (years)			0.614			0.901
<27	−21.79	[−85.43; 41.85]		7.79	[−58.33; 73.91]	
27–29	1			1		
29–33	4.42	[−57.82; 66.66]		16.44	[−44.65; 77.53]	
>33	19.89	[−43.38; 83.16]		23.01	[−40.84; 86.86]	
History of low birth weight			0.579			0.483
No	1			1		
Yes	−34.01	[−154.30; 86.28]		−43.64	[−165.54; 78.26]	
Household occupation			0.842			0.987
Advantageous	1			1		
Moderately advantageous	2.04	[−50.36; 54.45]		−3.68	[−55.72; 48.35]	
Disadvantageous	−18.74	[−97.09; 59.61]		−5.94	[−86.70; 74.83]	
Gestational diabetes			0.108			0.169
No	1			1		
Yes	63.25	[−13.97; 140.47]		53.76	[−22.95; 130.46]	
Rural location of residence at birth			0.911			0.722
No	1			1		
Yes	−2.57	[−47.72; 42.58]		−8.69	[−56.66; 39.28]	

95%CI: 95% confidence interval. ^&^ weighted analysis on the inverse of sampling probability.

**Table 3:** Comparison of non-nested models of fetal weight evolution according to active tobacco exposure and drinking water mixture exposure during pregnancy, Deux-Sèvres, France.

Comparison of Non-Nested Models of Fetal Weight Evolution according to Active Tobacco Exposure and Drinking Water Mixture Exposure during Pregnancy	Fetal Weight between Second Trimester and Birth in Grams ^&^ (*N* = 458)					
	Only Drinking-Water Exposure Model #2 AIC = 19,658.4			Only Tobacco Exposure Model #3 AIC = 19,636.7		
	Difference	95%CI	*p*	Difference	95%CI	*p*
Weight evolution according to mixture exposure in drinking water during pregnancy in grams			0.506			
Atrazine metabolites No and Nitrates < 18.14 mg/L	1					
Atrazine metabolites No and Nitrates 18.14–30.33 mg/L	−0.64	[−4.46; 3.19]				
Atrazine metabolites No and Nitrates > 30.33 mg/L	−1.52	[−6.00; 2.97]				
Atrazine metabolites Yes and Nitrates < 18.14 mg/L	1.93	[−2.02; 5.89]				
Atrazine metabolites Yes and Nitrates 18.14−30.33 mg/L	−0.03	[−4.61; 4.55]				
Atrazine metabolites Yes and Nitrates > 30.33 mg/L	0.98	[−3.01; 4.98]				
Weight evolution according to smoking during pregnancy						0.010
No				1		
Yes				−3.43	[−6.05; −0.82]	
Season during second trimester			0.779			0.776
Summer	1			1		
Fall	−31.44	[−91.73; 28.85]		−29.55	[−89.25; 30.15]	
Winter	−19.72	[−79.02; 39.58]		−21.55	[−79.65; 36.54]	
Spring	−13.96	[−76.89; 48.97]		−9.55	[−72.14; 53.04]	
Newborn sex			0.010			0.003
Boy	1			1		
Girl	−57.97	[−101.90; −14.05]		−65.23	[−108.87; −21.58]	
Maternal weight before pregnancy (kg)	2.51	[0.93; 4.09]	0.002	2.44	[0.87; 4.01]	0.002
Maternal age (years)			0.749			0.895
<27	−8.98	[−74.13; 56.17]		7.24	[−58.83; 73.30]	
27–29	1			1		
29–33	12.51	[−48.96; 73.98]		16.12	[−45.03; 77.27]	
>33	24.40	[−39.93; 88.73]		23.29	[−40.06; 86.64]	
History of low birth weight			0.340			0.450
No	1			1		
Yes	−59.38	[−181.49; 62.74]		−46.74	[−167.99; 74.51]	
Household occupation			0.862			0.998
Advantageous	1			1		
Moderately advantageous	−6.58	[−58.93; 45.77]		−0.58	[−52.38; 51.23]	
Disadvantageous	−22.24	[−102.38; 57.90]		−2.29	[−82.81; 78.23]	
Gestational diabetes			0.217			0.156
No	1			1		
Yes	48.63	[−28.54; 125.80]		55.25	[−21.10; 131.60]	
Rural location of residence at birth			0.791			0.692
No	1			1		
Yes	−6.51	[−54.81; 41.79]		−9.04	[−53.90; 35.82]	

95%CI: 95% confidence interval; AIC: Akaike criterion. ^&^ weighted analysis on the inverse of sampling probability.

## Data Availability

The datasets during and/or analyzed during the current study available from the corresponding author on reasonable request.
